# Supplementary material for: Men’s perceptions of long-term health outcomes following fertility problems: a UK-based mixed-methods survey
Source: J Assist Reprod Genet. 2026 Feb 27;43(5):1459–73. doi: 10.1007/s10815-026-03835-z (PMC13221544; doi:10.1007/s10815-026-03835-z)
Supplement: Supplementary file 1 — Supplementary Material 1 (DOCX 26.2 KB) [file 10815_2026_3835_MOESM1_ESM.docx]

**Supplementary Table 1** Association between men’s awareness of potential fertility-related adverse health outcomes (Q4) and other characteristics (Q1–3, 6, 9–15 and 19–20).

|  | | **Awareness of potential fertility-related adverse health outcomes (Q4)** | | **Group differences** |
| --- | --- | --- | --- | --- |
|  |  | **Yes** | **No** |  |
| **Domain** | **Characteristics** | ***n*** | ***n*** | ***p*-value** |
| **1** | **Age group (Q13)** |  |  | 0.250 |
|  | 18–34 | 1 | 7 |  |
|  | 35–39 | 1 | 9 |  |
|  | 40–44 | 8 | 11 |  |
|  | ≥45 | 5 | 15 |  |
|  | **Highest level of education (Q14)** |  |  | 0.679 |
|  | Finished Secondary school | 0 | 4 |  |
|  | Finished High School or College or equivalent | 3 | 9 |  |
|  | Further qualifications, a University Degree or higher | 12 | 29 |  |
|  | **Ethnicity (Q15)** |  |  | 0.739 |
|  | White (English, Irish, Scottish, etc.) | 12 | 33 |  |
|  | Asian (Indian, Pakistani, Chinese, etc.) | 1 | 3 |  |
|  | Black (Caribbean, African) | 2 | 2 |  |
|  | Mixed (White & Black Caribbean/Asian, etc.) | 0 | 1 |  |
|  | Other ethnic group (Arab, etc.) | 0 | 3 |  |
|  | **UK resident (Q16)** |  |  | **0.018*** |
|  | Yes | 7 | 32 |  |
|  | No | 5 | 3 |  |
| **2** | **ART status (Q1)** |  |  | 0.408 |
|  | Yes, undergone ART | 7 | 26 |  |
|  | Yes, considering ART | 5 | 8 |  |
|  | No | 9 | 20 |  |
|  | **ART timeframe (Q2)** |  |  | **0.015*** |
|  | <1 year ago | 7 | 8 |  |
|  | 1–3 years ago | 0 | 9 |  |
|  | 3–5 years ago | 0 | 2 |  |
|  | >5 years ago | 0 | 7 |  |
|  | **ART clinic type (Q3)** |  |  | 0.686 |
|  | NHS clinic | 3 | 9 |  |
|  | Private clinic | 4 | 17 |  |
|  | **Fertility evaluation performed before ART (Q9)** |  |  | 0.056 |
|  | Yes | 3 | 19 |  |
|  | No | 3 | 2 |  |
|  | **Fertility evaluation results discussed (Q10)** |  |  | 1.000 |
|  | Yes | 3 | 16 |  |
|  | No | 0 | 3 |  |
|  | **Fertility evaluation results indicated fertility problems (Q11)** |  |  | 1.000 |
|  | Yes | 3 | 12 |  |
|  | No | 0 | 4 |  |
|  | **Potential adverse health outcomes associated with fertility problems discussed (Q12)** |  |  | 0.081 |
|  | Yes | 2 | 1 |  |
|  | No | 1 | 11 |  |
| **3** | **Concerns about potential fertility-related adverse health outcomes (Q6)** |  |  | 0.122 |
|  | Yes | 15 | 34 |  |
|  | No | 2 | 18 |  |
| **4** | **Concerns about using confidential patient information without consent (Q17)** |  |  | 1.000 |
|  | Yes | 1 | 5 |  |
|  | No | 8 | 23 |  |

^*^ *^p^* ^< 0.05^

^Abbreviations: ART, assisted reproduction treatment; NHS, National Health Service; Q, question number; UK, United Kingdom^

**Supplementary Table 2** Association between men’s concerns about potential fertility-related adverse health outcomes (Q6) and other characteristics (Q1–4, 9–15 and 19–20).

|  | | **Concerns about potential fertility-related adverse health outcomes (Q6)** | | **Group differences** |
| --- | --- | --- | --- | --- |
|  |  | **Yes** | **No** |  |
| **Domain** | **Characteristics** | ***n*** | ***n*** | ***p*-value** |
| **1** | **Age group (Q13)** |  |  | 0.682 |
|  | 18–34 | 6 | 3 |  |
|  | 35–39 | 9 | 1 |  |
|  | 40–44 | 14 | 5 |  |
|  | ≥45 | 14 | 6 |  |
|  | **Highest level of education (Q14)** |  |  | 0.879 |
|  | Finished Secondary school | 3 | 1 |  |
|  | Finished High School or College or equivalent | 9 | 4 |  |
|  | Further qualifications, a University Degree or higher | 31 | 10 |  |
|  | **Ethnicity (Q15)** |  |  | **0.007*** |
|  | White (English, Irish, Scottish, etc.) | 36 | 9 |  |
|  | Asian (Indian, Pakistani, Chinese, etc.) | 3 | 2 |  |
|  | Black (Caribbean, African) | 4 | 0 |  |
|  | Mixed (White & Black Caribbean/Asian, etc.) | 0 | 1 |  |
|  | Other ethnic group (Arab, etc.) | 0 | 3 |  |
|  | **UK resident (Q16)** |  |  | 0.666 |
|  | Yes | 32 | 8 |  |
|  | No | 6 | 2 |  |
| **2** | **ART status (Q1)** |  |  | 0.579 |
|  | Yes, undergone ART | 24 | 8 |  |
|  | Yes, considering ART | 9 | 4 |  |
|  | No | 15 | 9 |  |
|  | **ART timeframe (Q2)** |  |  | **0.001*** |
|  | <1 year ago | 14 | 0 |  |
|  | 1–3 years ago | 7 | 2 |  |
|  | 3–5 years ago | 1 | 1 |  |
|  | >5 years ago | 2 | 5 |  |
|  | **ART clinic type (Q3)** |  |  | 0.433 |
|  | NHS clinic | 8 | 4 |  |
|  | Private clinic | 16 | 4 |  |
|  | **Fertility evaluation performed before ART (Q9)** |  |  | 1.000 |
|  | Yes | 18 | 4 |  |
|  | No | 4 | 1 |  |
|  | **Fertility evaluation results discussed (Q10)** |  |  | 1.000 |
|  | Yes | 15 | 4 |  |
|  | No | 3 | 0 |  |
|  | **Fertility evaluation results indicated fertility problems (Q11)** |  |  | 0.178 |
|  | Yes | 13 | 2 |  |
|  | No | 2 | 2 |  |
|  | **Potential adverse health outcomes associated with fertility problems discussed (Q12)** |  |  | 1.000 |
|  | Yes | 3 | 0 |  |
|  | No | 10 | 2 |  |
| **3** | **Awareness of potential fertility-related adverse health outcomes (Q4)** |  |  | 0.122 |
|  | Yes | 15 | 2 |  |
|  | No | 34 | 18 |  |
| **4** | **Concerns about using confidential patient information without consent (Q17)** |  |  | 0.309 |
|  | Yes | 6 | 0 |  |
|  | No | 24 | 8 |  |

^*^ *^p^* ^< 0.05^

^Abbreviations: ART, assisted reproduction treatment; NHS, National Health Service; Q, question number; UK, United Kingdom^
